# Relationship Between Obesity, Stress, and Age of Menarche Among Adolescent Female Students in Al-Madinah, Saudi Arabia

**DOI:** 10.7759/cureus.93739

**Published:** 2025-10-02

**Authors:** Magda H Youssef, Saada S Alharbi, Hoda A Alsubhi, Rawan A Almarwani, Ola A Farhan, Lama S Alahmadi, Amirah Aljohani, Rawan A Alharbi, Raghad A Almarwani, Rana M Arabi Katbi

**Affiliations:** 1 Physiology, Al-Rayan Colleges, Al-Madinah Al-Monawarrah, SAU; 2 Medicine and Surgery, Al-Rayan Colleges, Al-Madinah Al-Monawarrah, SAU; 3 Plastic and Burn Unit, Royal Commission Medical Center, Yanbu, SAU

**Keywords:** adolescents. percentage of early menarche, age of menarche, body mass index, obesity-stress correlation, saudi arabia, stress

## Abstract

Background

Menarche marks a pivotal milestone in a girl’s life, signaling the onset of puberty and triggering psychological and physiological changes. Recent evidence suggests that factors such as body weight and psychological stress may influence menarcheal timing, yet limited local data exist in Al-Madinah, Saudi Arabia.

Objective

This study aimed to determine relationships between obesity, perceived stress, and menarcheal age among middle school female students in Al-Madinah, Saudi Arabia, while investigating menstrual health patterns and regional variations.

Methods

A cross-sectional study was conducted among 349 randomly selected middle school female students in Al-Madinah between September 2023 and September 2024. Data collection involved self-administered questionnaires, including menstrual history and the Perceived Stress Scale (PSS). Anthropometric measurements (height, weight, waist circumference) were obtained, and body mass index (BMI) was calculated.

Results

Among participants, 4.9% were obese and 12.9% overweight. Early menarche (10-12 years) occurred in 74.8% of students, with a mean menarcheal age of 11.53 ± 1.6 years. High perceived stress affected 9.7% of students, with 23.5% of obese students experiencing high stress compared to 9% of non-obese students (p=0.049). A significant negative correlation existed between perceived stress and menarcheal age (r=−0.11, p=0.027). Extended menstrual flow (>7 days) occurred in 43.3% of students, premenstrual symptoms in 65%, and dysmenorrhea in 79.9%. High stress was associated with irregular cycles (p=0.005). Multivariate analysis identified perceived stress, marital status, and non-academic stressors as independent obesity predictors.

Conclusion

Higher perceived stress levels were significantly associated with earlier menarche among Al-Madinah adolescents. The study revealed complex relationships between stress, obesity, and menstrual health patterns, with notable regional variations. These findings highlight the need for integrated approaches addressing both physical and psychosocial factors in adolescent health programs.

## Introduction

The most important event that signifies the development of the female reproductive system is the menarche, or the start of menstruation. The scientific community acknowledges that a variety of factors, including body mass index (BMI), food, exercise, genetics, and environmental factors, influence the typical age at menarche [1]. With an average onset age of 12.4 years, menarche typically happens between the ages of 10 and 16 [2]. According to recent studies, the average age at menarche was 13.1 years. The menarche is a crucial time in a woman's life since it is a time of many physical and psychological changes that are essential to maintaining life behaviors [3].

One factor that is linked with menarche age is weight gain or obesity. Increased BMI is always strongly associated with an earlier menstrual age, according to a number of study platforms. Some research, however, contends that other factors may be at play and that obesity is not only caused by this later menarche age. Some contend that since the majority are mainly cross-sectional research, it is challenging to conclude causality from them. Other puberty-related indicators, like the growth of pubic and breast hair, are also linked to BMI [4].

BMI is a statistical measure that calculates body fat in men and women of any age based on height and weight. The output of the equation is then utilized to compute the BMI of the individual. The National Institute of Health (NIH) now uses BMI to categorize individuals as underweight, normal weight, overweight, or obese in place of traditional height vs. weight tables [5].

Without observing the individual, BMI cannot reveal the distribution of body fat. Compared to BMI, the waist-hip ratio (WHR) is simpler to compute and can provide more precise abdominal fat. It's interesting to note that in a big study conducted in the United States (US), participants' self-assessed BMI showed a strong correlation with specific biomarkers like triglycerides and leptin, but a weak correlation with more accurate classifications of obesity prevalence. At the very least, WHR and BMI were always computed in parallel for greater calculation accuracy [6]. BMI (kg/m2) had been used to measure overall obesity, whereas waist circumference and waist/hip ratio were used to measure central obesity (the storage of visceral adipose tissue) [7].

Higher rates of psychological issues in adulthood appear to be caused by post-pubertal stressful situations that are triggered by an earlier menarche. According to these findings, early growing girls' adolescent experiences steer them towards life trajectories where stress, hardship, and other threats to psychological health are more likely to persist in day-to-day existence [8]. Teenagers' association of menstruation with negative physical and psychological changes, which reflects misinformation, misperception, and the fear of being different from others, may be the cause of the physical and psychosocial issues that result from early menarche [9].

In general, precocious development of secondary sex characteristics increases the risk of emotional difficulty because it increases the likelihood that young people will encounter new stressors, norms, and expectations in their environments before they are psychologically ready to deal with them [10].

This study aimed to determine the relationship between obesity, perceived stress, and the age of menarche among middle school female students in Al-Madinah, Saudi Arabia. Specifically, it sought to assess menarcheal age, evaluate perceived stress levels, identify obesity prevalence, examine associations between obesity and stress with menarche onset, and investigate menstrual health patterns, including cycle regularity, flow duration, premenstrual symptoms, and dysmenorrhea. Additionally, the study explored regional variations in menstrual health outcomes and identified predictors of obesity. No previous local research has addressed these comprehensive relationships among adolescent girls in Al-Madinah, despite this developmental stage being critical for physical and psychological health. This study represents an initial step toward building local evidence to inform preventive strategies and reproductive health programs targeting adolescent girls.

## Materials and methods

Study design, study area, and time

A cross-sectional study of 349 female middle school students was randomly selected from government schools in Al-Madinah, Saudi Arabia, and conducted from September 2023 to September 2024. Al-Madinah is the second-holiest city for Muslims; a total of 36,414 female students are enrolled in 119 governmental middle schools for females in Al-Madinah.

Study population

Female adolescents between the ages of 13 and 16 who are enrolled in governmental middle schools and who are willing to engage in this study are the participants who match the inclusion criteria. Exclusion criteria: students with long-term medical conditions, mental health conditions, pathologically early menarche (menarche before the age of 8), medication use, positive pregnancy tests, and lactation were not allowed to participate in this study.

Sample size

The predetermined sample size was predicted to be 349, based on the results of a previous study with 35.6% of early menarche [3]. Calculated using the OpenEpi website software formula, version 3, open-source calculator using the equation {Sample size n = [DEFF*Np(1-p)]/ [(d2/Z21-α/ 2*(N-1) +p*(1-p)]} [10]. Through a 5% margin of error, a 95% confidence interval (CI), and a 10% increase in compensation to make up for the incomplete survey.

Sampling technique

‏Schools were chosen through a multistage selection process. A list of all governmental middle schools in Al-Madinah was obtained from the Ministry of Education. From a total of 119 schools, 15 were selected. In each selected school, one class from each grade level was randomly chosen, and all eligible students in those classes were invited to participate in the study.

Data collection

Data for this study were collected directly by the research investigators. Anthropometric measurements were obtained by the research team, while self-administered questionnaires were completed by participants under the supervision and presence of the investigators. The anthropometric measurements included weight (measured in kilograms), height, and waist circumference (both measured in centimeters). BMI was calculated by dividing each participant’s weight in kilograms by height in meters squared. BMI values were subsequently converted to age-specific percentiles using the BMI-for-age growth charts for children and adolescents (ages 2-20 years) provided by the Centers for Disease Control and Prevention [11]. Weight classification was determined as follows: students below the fifth percentile were classified as underweight, those between the fifth and 85th percentiles as normal weight, those between the 85th and 95th percentiles as overweight, and those at or above the 95th percentile as obese. The self-administered questionnaire, originally developed in English, was translated into Arabic by the research investigators [3] and thoroughly reviewed by subject matter experts to ensure face validity. A pilot study involving 20 students was conducted to verify the clarity and cultural appropriateness of the translated instrument. The questionnaire was designed to collect comprehensive information regarding demographic characteristics, age at menarche, menstrual patterns, characteristics of menstrual blood loss, history of abnormal vaginal bleeding or intermenstrual spotting, premenstrual symptoms, and dysmenorrhea history. The Perceived Stress Scale (PSS), a validated instrument used to assess psychological stress experienced by individuals during the preceding month [12], was also included in the study protocol.​​

Ethical consideration

Signed informed consent was obtained from the student's parents, ensuring that they are fully informed about the study's objectives and benefits. Participants had the right to withdraw from the study at any time without any consequences. Ethical approval of the research was obtained from the ethical committee in Al-Rayan Colleges, Al-Madinah, Saudi Arabia, with ID number (HA-03-M-122-055). The confidentiality of the participants' information was strictly maintained throughout the study. Data were stored securely and were only accessible by authorized research personnel. Any identifying information was anonymized or removed to ensure participant privacy and confidentiality. Participation in the study was completely voluntary. Participants were informed that their involvement was not obligatory, and they had the freedom to decline or withdraw without any negative consequences or impact on their academic standing. The study was conducted with the utmost consideration for the physical and psychological well-being of the participants. Any potential risks associated with discussing sensitive topics or personal experiences were minimized by providing appropriate support, resources, and referrals to counseling services or relevant healthcare providers, if necessary. These ethical considerations are aimed at protecting the rights, privacy, and well-being of the participants and ensuring that the study is conducted ethically and with integrity

Data analysis

Data were statistically analyzed using SPSS version 26 (IBM Corp., Armonk, New York, USA). To investigate the association between the variables, the Chi-squared test (χ2) was applied to qualitative data that was expressed as numbers and percentages. The association between the quantitative non-parametric variables that were expressed as mean and standard deviation (Mean ± SD) was examined using the Kruskal-Wallis test for non-parametric variables. Correlation analysis was performed using the Spearman's test and statistical significance was defined as a p-value of less than 0.05 was considered statistically significant. Multivariate logistic regression analysis was done to assess factors associated with obesity and stress among the studied participants. The odds ratio (OR) was calculated at a 95% CI.

## Results

Of the studied participants and based on the BMI classification, 17 students (2.9%) were underweight, 270 (77.4%) had normal weight, 45 (12.9%) were overweight, and 17 (4.9%) were obese. The mean BMI for the previous BMI categories was: 14.06 ± 0.74, 19,92 ± 2.91, 26.97 ± 1.55, and 34.17 ± 3.2 km/m2, respectively, as shown in Table 1.

**Table 1:** Comparison between obese and none obese students according to their demographics and academic year (No.: 349) * = Kruskal Wallis test; SD = standard deviation.

Variable	Total	Obesity				χ2	p-value
	M ±SD	Underweight M. ±SD	Normal weight M ±SD	Overweight M. ±SD	Obese M. ±SD		
Age (years) (Mean ± SD)	13.9 ±1.2	13.716 ± 1.35	13.86 ±1.17	13.91 ± 1.37	14.65 ±0.99	3*	0.05
Marital status	No. (%)	No. (%)	No. (%)	No. (%)	No. (%)	32.21	<0.01
Single	338 (96.8)	17 (100)	267 (98.9)	41 (91.1)	13 (76.5)		
Married	11 (3.2)	0 (0.0)	3 (1.1)	4 (8.9)	4 (23.5)		
Academic year	No. (%)	No. (%)	No. (%)	No. (%)	No. (%)	10.47	0.106
1^st^ grade	84 (24.1)	7 (41.2)	69 (25.6)	8 (17.8)	0 (0.0)		
2^nd^ grade	121 (34.7)	5 (29.4)	93 (34.4)	17 (37.8)	6 (35.3)		
3^rd^ grade	144 (41.3)	5 (29.4)	108 (40)	20 (44.4)	11 (64.7)		

Obesity results

Of the studied participants, 17 (2.9%) were obese, with a mean BMI of 21.24 ± 4.85. The mean age of the studied students was 13.9±1.2 years, 3.2% were married, and 42.3% were in the 3rd grade. On comparison between BMI categories, it was found that obese students had a significantly higher percentage of being married (p=0.05), as shown in Table 1.

Table 2 shows that 74.8% of students had an early menarche (10 to 12 years) with a mean menarche age of 11.53 ± 1.6 years. Of students, 43.3% had a duration of flow of more than seven days, and 60.7% had regular menstruation. Of them, 24.4% had a history of amenorrhea, and 59.6% had an average (blood loss of 50 to 80, five to seven pads/day). For those with abundant blood loss, 59.1% reported soaking through one or more sanitary pads every hour for several consecutive hours. Only 3.7% had any history of abnormal vaginal bleeding; of them, 38.8% reported having polycystic ovarian disease. It was found that obese students had a significantly higher percentage of having an early menarche age and having polycystic ovarian disease, and underweight students had a significantly higher percentage of having a history of abnormal vaginal bleeding or spotting between periods(p<0.05). On the other hand, a non-significant difference was found between BMI categories according to menstrual pattern (p>0.05).

**Table 2:** Comparison between obese and none obese students according to menarche age, menstrual pattern and history of abnormal vaginal bleeding (No.: 349) * = Kruskal Wallis test; DM = diabetes mellitus.

Variable	Total	Obesity				χ2	p-value
	No. (%)	Underweight No. (%)	Normal weight No. (%)	Overweight No. (%)	Obese No. (%)		
Menarche age (years)						15.48	0.017
Early menarche (10 to 12)	261 (74.8)	8 (47.1)	209 (77.4)	31 (68.9)	13 (76.5)		
Middle menarche (13 to 14)	83 (23.8)	9 (52.9)	59 (21.9)	12 (26.7)	3 (17.6)		
Late menarche (15 to 16)	5 (1.4)	0 (0.0)	2 (0.7)	2 (4.4)	1 (5.9)		
Menarche age (years) (Mean ± SD)	11.53 ± 1.6	11.88 ± 2.49	11.49 ± 1.45	11.87 ± 1.56	10.82 ± 2.55	3*	0.052
Duration of flow in days (in the last three months)						6.47	0.372
Less than 4	54 (15.5)	3 (17.6)	37 (13.7)	12 (26.7)	2 (11.8)		
5 to 6	144 (41.3)	5 (29.4)	117 (43.3)	15 (33.3)	7 (41.2)		
More than 7	151 (43.3)	9 (52.9)	116 (43)	18 (40)	8 (47.1)		
Menstrual regularity (in the last three months)						2.33	0.507
Irregular	137 (39.3)	7 (41.2)	110 (40.7)	13 (28.9)	7 (41.2)		
Regular	212 (60.7)	10 (58.8)	160 (59.3)	32 (71.1)	10 (58.8)		
History of amenorrhea (absent menstrual period since the last three months, if you were previously menstruating normally)						0.89	0.826
No	264 (75.6)	12 (70.6)	207 (76.7)	32 (71.1)	13 (76.5)		
Yes	85 (24.4)	5 (29.4)	63 (23.3)	13 (28.9)	4 (23.5)		
Characteristics of menstrual blood loss (in the last three months)						10.07	0.121
Scares (blood loss of 1 to 49ml, ≤4 pads/day)	119 (34.1)	6 (35.3)	90 (33.3)	21 (46.7)	2 (11.8)		
Average (blood loss of 50 to 80, 5-7 pads/day)	208 (59.6)	9 (52.9)	166 (61.5)	20 (44.4)	13 (76.5)		
Abundant (blood loss greater than 80ml, ≥8 pads/day)	22 (6.3)	2 (11.8)	14 (5.2)	4 (8.9)	2 (11.8)		
If the blood loss is abundant, more than 80ml, is it associated with: (No.: 208)							
Soaking through one or more sanitary pads every hour for several consecutive hours	13 (59.1)	1 (33.3)	10 (71.4)	2 (50)	0 (0.0)	3.28	0.35
Needing to use double sanitary protection to control your menstrual flow	6 (28.6)	1 (33.3)	4 (30.8)	1 (25)	0 (0.0)	4.94	0.551
Needing to wake up to change sanitary protection during the night	9 (40.9)	2 (66.7)	5 (35.7)	1 (25)	1 (100)	2.84	0.416
Passing large blood clots with menstrual flow	4 (18.2)	1 (33.3)	1 (7.1)	2 (50)	0 (0.0)	4.55	0.208
Restricting daily activities due to heavy menstrual flow	3 (14.3)	0 (0.0)	3 (21.4)	0 (0.0)	0 (0.0)	1.98	0.576
Symptoms of anemia, such as tiredness, fatigue or shortness of breath	3 (14.3)	0 (0.0)	2 (15.4)	1 (25)	0 (0.0)	1.05	0.788
Is there any history of abnormal vaginal bleeding or spotting between periods?						14.01	0.003
No	336 (96.3)	14 (82.4)	264 (97.8)	43 (95.6)	15 (88.2)		
Yes	13 (3.7)	3 (17.6)	6 (2.2)	2 (4.4)	2 (11.8)		
If yes, do you have a history of: (No.: 13)						42.81	<0.001
Polycystic ovarian disease	5 (38.8)	0 (0.0)	2 (0.7)	1 (2.2)	2 (11.8)		
Hormonal family planning	1 (7.6)	0 (0.0)	1 (0.4)	0 (0.0)	0 (0.0)		
Pelvic inflammatory disease	2 (15.3)	1 (5.9)	1 (0.4)	0 (0.0)	0 (0.0)		
Taking any other medicine	4 (30.7)	2 (11.8)	1 (0.4)	1 (2.2)	0 (0.0)		
Any other associated illness: DM	1 (7.6)	0 (0.0)	1 (0.4)	0 (0.0)	0 (0.0)		

Table 3 shows that 65% of students had premenstrual symptoms, with cramps (61.2%), backache (59%), and tiredness (58.1%) the most common symptoms. The majority (71.9%) had no symptoms of water retention, and most of the students (72.2%) reported having mood swings as negative effects. The majority (79.9%) had a history of dysmenorrhea; of them, 60.2% had pain in both the lower abdomen and back, and 22.5% reported a high effect of pain on their working ability. It was found that obese students had a significantly higher percentage of having any of the water retention symptoms, especially tender, compared with students with other BMI categories (p<0.05).

**Table 3:** Comparison between obese and none obese students according to history of premenstrual symptoms and dysmenorrhea (No.: 349)

Variable	Total	Obesity				χ2	p-value
	No. (%)	Underweight No. (%)	Normal weight No. (%)	Overweight No. (%)	Obese No. (%)		
Are your menstrual cycles associated with premenstrual symptoms						1.48	0.686
No	122 (35)	4 (23.5)	98 (36.3)	14 (31.1)	6 (35.3)		
Yes	227 (65)	13 (76.5)	172 (73.7)	31 (68.9)	11 (64.7)		
If yes, do you feel any of the following: (No.: 227)							
Generalized Pains	111 (48.9)	7 (53.8)	83 (48.3)	16 (51.6)	5 (45.5)	0.29	0.96
Cramps	139 (61.2)	9 (69.2)	103 (59.9)	20 (64.5)	7 (63.6)	0.65	0.885
Acne	119 (52.4)	8 (61.5)	92 (53.5)	13 (41.9)	6 (54.5)	1.89	0.584
Backache	134 (59)	9 (69.2)	100 (58.1)	17 (54.8)	8 (72.7)	1.69	0.638
Headache	96 (42.3)	9 (69.2)	68 (39.5)	13 (41.9)	6 (54.5)	5.08	0.166
Tiredness	132 (58.1)	7 (53.8)	99 (57.6)	21 (67.7)	5 (45.5)	2.02	0.567
Water retention						23.92	0.004
Tender breasts	40 (11.5)	3 (17.6)	23 (8.5)	8 (17.8)	6 (35.3)		
Bloating	31 (8.9)	0 (0.0)	26 (9.6)	4 (8.9)	1 (5.9)		
Both	27 (7.7)	0 (0.0)	18 (6.7)	6 (13.3)	3 (17.6)		
None	251 (71.9)	14 (82.4)	203 (75.2)	27 (60)	7 (41.2)		
Negative effects							
Loss of appetite	124 (42)	8 (50)	99 (43.8)	16 (41)	1 (7.1)	7.71	0.052
Anger	170 (57.6)	9 (56.3)	129 (57.1)	24 (61.5)	8 (57.1)	0.28	0.963
Mood swings	213 (72.2)	14 (87.5)	159 (70.4)	30 (76.9)	10 (71.4)	2.68	0.442
History of dysmenorrhea (Pain during menstruation)						3.51	0.319
No	70 (20.1)	2 (11.8)	60 (22.2)	6 (13.3)	2 (11.8)		
Yes	279 (79.9)	15 (88.2)	210 (77.8)	39 (86.7)	15 (88.2)		
If yes, identify the pain location (No.: 279)							
Lower abdomen	146 (52.3)	10 (66.7)	113 (53.8)	19 (48.7)	4 (26.7)	5.58	0.134
Lower abdomen and back	168 (60.2)	7 (46.7)	127 (60.5)	22 (56.4)	12 (80)	3.84	0.279
Right side of the abdomen	39 (14)	3 (20)	25 (11.9)	8 (20.5)	3 (20)	3.04	0.385
Left side of the abdomen	33 (11.8)	3 (20)	21 (10)	7 (17.9)	2 (13.3)	3.06	0.381
Does the pain affect s on your working ability? (No.: 279)						19.81	0.084
High effect	63 (22.5)	1 (5.9)	52 (19.3)	7 (15.6)	3 (17.6)		
Moderate effect	90 (32.5)	5 (29.4)	74 (27.4)	8 (17.8)	3 (17.6)		
Low effect	68 (24.3)	5 (29.4)	49 (18.1)	9 (20)	5 (29.4)		
Not effected	58 (20.7)	4 (23.5)	35 (13)	15 (33.3)	4 (23.5)		

Table 4 shows that 42.1% of students reported being stressed sometimes, while 9.7% reported always being stressed. The most common stressors were related to studies (44.1%). Based on the PSS score classification, 9.7% of students had a high perceived stress, while 74.8% had moderate stress, and 15.5% had a low stress level. It was found that obese students had a significantly higher percentage of having stressors other than those related to study, exams, or social stressors (p <0.05).

**Table 4:** Comparison between obese and none obese students according to history of stress, stressors and stress level based on the Perceived Stress Scale score classification (No.: 349)

Variable	Total	Obesity				χ2	p-value
	No. (%)	Underweight No. (%)	Normal weight No. (%)	Overweight No. (%)	Obese No. (%)		
Are you stressed most of the time						6.03	0.914
Never	65 (18.6)	2 (11.8)	52 (19.3)	8 (17.8)	3 (17.6)		
Rarely	55 (15.8)	3 (17.6)	43 (15.9)	4 (8.9)	5 (29.4)		
Sometimes	147 (42.1)	8 (47.1)	114 (42.2)	20 (44.4)	5 (29.4)		
Often	48 (13.8)	3 (17.6)	35 (13)	8 (17.8)	2 (11.8)		
Always	34 (9.7)	1 (5.9)	26 (9.6)	5 (11.1)	2 (11.8)		
What are your identified stress factors?							
Related to studies	154 (44.1)	8 (47.1)	121 (44.8)	22 (48.9)	3 (17.6)	5.36	0.147
Related to the exam	178 (51)	8 (47.1)	140 (51.9)	20 (44.4)	10 (58.8)	1.37	0.712
Social stress	121 (34.7)	7 (41.2)	94 (34.8)	15 (33.3)	5 (29.4)	0.56	0.905
Other types of stress	105 (30.1)	5 (29.4)	78 (28.9)	11 (24.4)	11 (64.7)	10.55	0.014
Stress level based on the Perceived Stress Scale score classification						7.84	0.25
Low stress	54 (15.5)	4 (23.5)	44 (16.3)	6 (13.3)	0 (0.0)		
Moderate stress	261 (74.8)	12 (70.6)	200 (74.1)	36 (80)	13 (76.5)		
High perceived stress	34 (9.7)	1 (5.9)	26 (9.6)	3 (6.7)	4 (23.5)		

Table 5 shows that obese students had a significantly higher percentage of having high perceived stress compared to non-obese students (23.5% vs. 9%) (p<0.05).

**Table 5:** Relationship between obesity and perceived stress

Variable	Non obese	Obese	χ2	p-value
	No. (%)	No. (%)		
Low or moderate stress	302 (91)	13 (76.5)	3.86	0.049
High perceived stress	30 (9)	4 (23.5)		

Table 6 shows that a significant positive correlation was found between BMI and students ‘age (r=0.11, p-value=0.037). On the other hand, a non-significant negative correlation was found between BMI and menarche age, duration of flow in days (in the last three months), and the PSS score (p>0.05).

**Table 6:** Spearman’s correlation analysis between body mass index (BMI) and students, age, menarche age, duration of flow in days (in last three months) and the Perceived Stress Scale score

Variable	BMI	
	r	p-value
Age	0.11	0.037
Menarche age	-0.07	0.16
Duration of flow in days (in the last three months)	-0.03	0.518
Perceived Stress Scale score	-0.06	0.235

Multivariate logistic regression analysis was done to assess the risk factors (independent predictors) of obesity among the studied students. It was found that being married, having stressors other than those related to study, exams, or social stressors, and having a high perceived stress level were risk factors (independent predictors) of obesity among the studied students (Table 7).

**Table 7:** Multivariate logistic regression analysis of risk factors (independent predictors) of obesity among studied students

Variable	B	Wald	p-value	Odds Ratio (CI:95%)
Marital status	3.45	14.41	0.001	1.03 (1.34- 2.55)
Menarche age	0.52	0.8	0.369	0.59 (0.18-1.86)
Polycystic ovarian disease	0.05	0.17	0.23	0.15 (0.33-1.28)
Water retention	0.53	6.12	0.013	1.85 (1.38-4.89)
Other type of stress	2.02	10.53	0.002	1.13 (1.03-3.45)
Stress level based on the Perceived Stress Scale score classification	1.42	5.95	0.015	4.15 (1.32-13.02)

Stress results

Table 8 demonstrates that the prevalence of high perceived stress was significantly higher among students in the 3rd grade (p<0.05).

**Table 8:** Comparison between stress levels and obese according to their demographics and academic year (No.: 349) * = Kruskal Wallis test; SD = standard deviation.

Variable	Stress level based on the Perceived Stress Scale score classification			χ2	p-value
	Low stress No. (%)	Moderate stress No. (%)	High perceived stress No. (%)		
Age (years) (Mean ± SD)	13.81 ± 1.29	13.85 ± 1.18	14.35 ± 1.22	2*	0.073
Marital status				0.37	0.827
Single	43 (98.1)	252 (96.6)	33 (97.1)		
Married	1 (1.9)	9 (3.4)	1 (2.9)		
Academic year				10.87	0.028
1^st^ grade	17 (1.5)	63 (24.1)	4 (11.8)		
2^nd^ grade	13 (24.1)	99 (37.9)	9 (26.5)		
3^rd^ grade	24 (44.4)	99 (37.9)	21 (61.8)		

Table 9 shows that the prevalence of high perceived stress was significantly higher among students who had irregular menstruation (p<0.05). On the other hand, a non-significant relationship was found between stress levels and menarche age, other menstrual patterns, or history of abnormal vaginal bleeding (p>0.05).

**Table 9:** Comparison between stress levels according to menarche age, menstrual pattern and history of abnormal vaginal bleeding (No.: 349)

Variable	Stress level based on the Perceived Stress Scale score classification			χ2	p-value
	Low stress No. (%)	Moderate stress No. (%)	High perceived stress No. (%)		
Menarche age				1.62	0.804
Early menarche (10 to 12)	41 (75.9)	192 (73.6)	28 (82.4)		
Middle menarche (13 to 14)	12 (22.2)	65 (24.9)	8 (17.6)		
Late menarche (15 to 16)	1 (1.9)	4 (1.5)	0 (0.0)		
Duration of flow in days (in the last three months)				5.27	0.261
Less than 4	8 (14.8)	44 (16.9)	2 (5.9)		
5 to 6	21 (38.9)	111 (42.5)	12 (35.3)		
More than 7	25 (46.3)	106 (40.6)	20 (58.8)		
Menstrual regularity (in the last three months)				10.71	0.005
Irregular	12 (22.2)	106 (40.6)	19 (55.9)		
Regular	42 (77.8)	155 (59.4)	15 (44.1)		
History of amenorrhea (absent menstrual period since the last three months, if you were previously menstruating normally)				4.91	0.085
No	46 (85.2)	196 (75.1)	22 (64.7)		
Yes	8 (14.8)	65 (24.9)	12 (35.3)		
Characteristics of Menstrual blood loss (in the last three months)				1.54	0.819
Scares (blood loss of 1 to 49ml, ≤4 pads/day)	18 (33.3)	87 (33.3)	14 (41.2)		
Average (blood loss of 50 to 80, 5-7 pads/day)	33 (61.1)	158 (60.5)	17 (50)		
Abundant (blood loss greater than 80ml, ≥8 pads/day)	3 (5.6)	16 (6.1)	3 (8.8)		
If the blood loss is abundant, more than 80ml, is it associated with: (No.: 208)					
Soaking through one or more sanitary pads every hour for several consecutive hours	2 (40)	10 (62.5)	1 (100)	1.52	0.467
Needing to use double sanitary protection to control your menstrual flow	1 (25)	5 (31.3)	0 (0.0)	0.9	0.924
Needing to wake up to change sanitary protection during the night	2 (40)	7 (43.8)	0 (0.0)	0.74	0.688
Passing large blood clots with menstrual flow	0 (0.0)	4 (25)	0 (0.0)	1.83	0.4
Restricting daily activities due to heavy menstrual flow	1 (20)	2 (12.5)	0 (0.0)	0.34	0.841
Symptoms of anemia, such as tiredness, fatigue, or shortness of breath	1 (25)	2 (12.5)	0 (0.0)	0.58	0.747
History of abnormal vaginal bleeding or spotting between periods				0.97	0.613
No	53 (98.1)	251 (96.2)	32 (94.1)		
Yes	1 (1.9)	10 (3.8)	2 (5.9)		
If yes, do you have a history of: (No.: 23)				12.26	0.267
Polycystic ovarian disease	0 (0.0)	4 (1.5)	1 (2.9)		
Hormonal family planning	0 (0.0)	1 (0.4)	0 (0.0)		
Pelvic inflammatory disease	0 (0.0)	2 (0.8)	0 (0.0)		
Taking any other medicine	1 (1.9)	3 (1.1)	0 (0.0)		
Any other associated illness: DM	0 (0.0)	0 (0.0)	1 (2.9)		

Table 10 shows that the prevalence of high perceived stress was significantly higher among students who suffered acne as a premenstrual symptom (p<0.05). while a non-significant relationship was found between stress levels and other premenstrual symptoms or dysmenorrhea (p>0.05).

**Table 10:** Comparison between stress levels according to history of premenstrual symptoms and dysmenorrhea (No.: 349)

Variable of the history of premenstrual symptoms	Stress level based on the Perceived Stress Scale score classification			χ2	p-value
	Low stress No. (%)	Moderate stress No. (%)	High perceived stress No. (%)		
Are your menstrual cycles associated with premenstrual symptoms				1.28	0.527
No	22 (40.7)	90 (34.5)	10 (29.4)		
Yes	32 (59.3)	171 (65.5)	24 (70.6)		
If yes, do you feel any of the following: (No.: 227)					
Generalized Pains	11 (34.4)	87 (50.9)	13 (54.2)	3.23	0.198
Cramps	17 (53.1)	110 (64.3)	12 (50)	2.85	0.24
Acne	12 (37.5)	90 (52.6)	17 (70.8)	6.12	0.047
Backache	15 (46.9)	103 (60.2)	16 (66.7)	2.63	0.268
Headache	10 (31.3)	77 (45)	9 (37.5)	2.34	0.309
Tiredness	13 (40.6)	105 (61.4)	14 (58.3)	4.78	0.092
Water retention				10.04	0.123
Tender breasts	2 (3.7)	35 (13.4)	3 (8.8)		
Bloating	2 (3.7)	24 (9.2)	5 (14,.7)		
Both	3 (5.6)	20 (7.7)	4 (11.8)		
None	47 (87)	182 (69.7)	22 (64.7)		
Negative effects					
Loss of appetite	15 (38.5)	91 (40.9)	18 (56.3)	3.04	0.219
Anger	17 (43.6)	133 (59.4)	20 (62.5)	3.73	0.154
Mood swings	26 (66.7)	161 (71.9)	26 (81.3)	1.91	0.284
History of dysmenorrhea (pain during menstruation)				1.15	0.562
No	13 (24.1)	52 (19.9)	5 (14.7)		
Yes	41 (75.9)	209 (80.1)	29 (85.3)		
If yes, identify the pain location (No.: 279)					
Lower abdomen	22 (53.7)	114 (54.5)	10 (34.5)	4.14	0.126
Lower abdomen and back	20 (48.8)	127 (60.8)	21 (72.4)	4.06	0.131
Right side of the abdomen	5 (12.2)	28 (13.4)	6(20.7)	1.25	0.534
Left side of the abdomen	4 (9.8)	23 (11)	6 (20.7)	2.48	0.288
Does the pain affect s on your working ability? (No.: 279)				11.57	0.171
High effect	4 (7.4)	50 (19.2)	9 (26.5)		
Moderate effect	10 (18.5)	69 (26.4)	11 (32.4)		
Low effect	14 (25.9)	49 (18.8)	5 (14.7)		
Not effected	13 (24.1)	41 (15.7)	4 (11.8)		

Table 11 shows that the prevalence of high perceived stress was significantly higher among students who reported always being stressed most of the time, and who had social stressors (p<0.05).

**Table 11:** Comparison between stress levels according to history of stress, stressors and stress level based on the Perceived Stress Scale score classification (No.: 349)

Variable	Stress level based on the Perceived Stress Scale score classification			χ2	p-value
	Low stress No. (%)	Moderate stress No. (%)	High perceived stress No. (%)		
Are you stressed most of the time				59.54	<0.001
Never	23 (42.6)	41 (15.7)	1 (2.9)		
Rarely	12 (22.2)	40 (15.3)	3 (8.8)		
Sometimes	17 (31.5)	120 (46)	10 (29.4)		
Often	1 (1.9)	38 (14.6)	9 (26.5)		
Always	1 (1.9)	22 (8.4)	11 (32.4)		
What are your identified stress factors?					
Related to studies	20 (37)	113 (43.3)	21 (61.8)	5.46	0.065
Related to the exam	28(51.9)	128 (49)	22 (64.7)	2.97	0.226
Social stress	13 (24.1)	91 (34.9)	17 (50)	6.2	0.045
Other type of stress	16 (29.6)	77 (29.5)	12 (35.3)	0.48	0.784

Table 12 shows that a significant negative correlation was found between the PSS score and menarche age (r=-0.11, p-value=0.027). On the other hand, a non-significant positive correlation was found between the PSS score and students’ age and duration of flow in days (in the last three months) (p>0.05). and a non-significant negative correlation was found between the PSS score and BMI (p>0.05).

**Table 12:** Spearman’s correlation analysis between stress level based on the Perceived Stress Scale score classification and students, age, menarche age, duration of flow in days (in last three months) and the Perceived Stress Scale score

Variable	Perceived Stress Scale score	
	r	p-value
Age	0.1	0.057
Menarche age	-0.11	0.027
Duration of flow in days (In last three months)	0.03	0.544
BMI	-0.06	0.235

Multivariate logistic regression analysis was done to assess the risk factors (independent predictors) of perceived stress among the studied students. It was found that having an early menarche (10 to 12), having an irregular menstrual cycle, and being always stressed most of the time were risk factors (independent predictors) of perceived stress among the studied students Table 13.

**Table 13:** Multivariate logistic regression analysis of risk factors (independent predictors) of perceived stress among studied students

Variable	B	Wald	p-value	Odds Ratio (CI:95%)
Academic year	0.59	3.09	0.079	0.8 (0.93-1.48)
Menstrual regularity (In last three months)	0.91	4.2	0.04	2.5 (1.04-6.03)
Menarche age	0.25	4.39	0.036	1.77 (1.61-3.98)
Menstrual cycles associated with acne as a premenstrual symptom	0.84	3.12	0.077	0.42 (0.16-1.09)
If stressed most of the time	0.93	9.85	<0.001	2.55 (1.16-4.56)
Social stress	0.2	0.21	0.64	0.81 (0.33-1.94)

## Discussion

This study assessed obesity, stress, and menarche age among Saudi Arabian adolescent female students in Al-Madinah. We included a total of 349 students, with 74.8% experiencing early menarche and a mean menarcheal age of 11.53 ±1.6 years. The distribution across academic levels was 24.1% in 1st grade, 34% in 2nd grade, and 41.3% in 3rd grade. The observed mean menarcheal age aligns with worldwide patterns showing a decrease in menarche age over recent decades. Our findings were lower compared to studies conducted in Dammam (13.1 years), Makkah (12.9 years), and Riyadh (13.08 years) [3], which have been linked to various factors, including psychological effects, physical activity levels, and nutritional health. However, our results are consistent with a 2018 Saudi Arabian study reporting a mean age of 12.46 ± 1.57 years [13].

Our study indicated that 12.9% of Al-Madinah high school girls were overweight and 4.9% were obese. These results align with studies conducted in Northern Saudi Arabia showing 16.3% of Saudi Arabian high school girls are obese or overweight [14]. This uniformity across locations reflects a broader trend in overweight and obesity prevalence among adolescent girls in Saudi Arabia. Regarding the correlation between menarcheal age and BMI, our findings indicate a non-significant negative relationship, suggesting that earlier onset of menarche may be associated with higher BMI, although this association did not reach statistical significance. This observation aligns with previous research reporting a significant negative correlation between menarcheal age and BMI [3]. Al-Madinah students demonstrated considerably higher prevalence of extended menstrual flow (>7 days) (43.3%) compared to Jeddah counterparts (29.3%) and significantly higher prevalence of premenstrual symptoms (65%) versus Jeddah students (41.5%) [15]. This disparity indicates geographical differences in menstrual health that may be influenced by lifestyle, diet, socioeconomic level, and access to medical care. The prevalence of dysmenorrhea among Al-Madinah students was 79.9%, comparable to but somewhat lower than the Jeddah study (84.8%) [15].

The study reveals complex interactions between menarche and stress among adolescent females. Early menarche (ages 10-12) was common, representing 74.8% of participants. Perceived stress was relatively high, with 9.7% experiencing high stress and 74.8% having moderate levels. A significant negative correlation was identified between PSS score and menarche age (r=-0.11, p=0.027), indicating that higher perceived stress levels are associated with earlier menarcheal onset. The physiological mechanism involves cortisol release during stressful situations, which triggers insulin, leptin, and neuropeptide-Y (NPY) system activation, stimulating hunger and eating behaviors. This aligns with studies showing that students under ten years are 1.19 times more likely to experience early menarche with high psychological stressors [16, 17].

Our findings highlight a significant association between high perceived stress and obesity. Multivariate analysis identified high perceived stress, marital status, and non-academic stressors as independent predictors of obesity. While direct correlation between stress and BMI was not significant (r=-0.06, p=0.235), logistic regression demonstrated perceived stress as a stronger risk factor when combined with specific health outcomes. This emphasizes that understanding stress severity, rather than frequency alone, is crucial for obesity prevention strategies [18]. Students in higher grades, specifically 3rd grade, exhibited a higher prevalence of high perceived stress (p<0.05), suggesting that academic or age-related pressures contribute to stress during late adolescence. Stress levels significantly influenced menstrual regularity, with high stress associated with irregular cycles (p=0.005). Premenstrual symptoms, particularly acne, correlated with higher stress levels (p=0.047), indicating psychosomatic relationships between psychological and physical changes during adolescence.

This study has several limitations that should be acknowledged. The cross-sectional design restricts the ability to establish causal relationships, capturing only a temporal snapshot rather than observing changes over time. Self-reported data on stress levels and menstrual history may introduce recall bias, potentially affecting finding accuracy. The study’s geographic and cultural specificity to Al-Madinah, Saudi Arabia, may limit generalizability to populations with different cultural, environmental, and lifestyle factors. Additionally, BMI measurements do not account for body composition differences such as muscle mass, which could influence the interpretation of findings.

Future research should adopt longitudinal designs to track changes over time and establish causal relationships between obesity, stress, and menarcheal age. Expanding research to include diverse populations from different regions and cultural backgrounds would enhance result generalizability. Investigating dietary habits, physical activity, and genetic predispositions could provide a more nuanced understanding of factors influencing early menarche. Integration of qualitative methods to explore psychosocial impacts of early menarche would offer deeper perspectives on emotional and social challenges. Finally, examining intervention program effectiveness focused on healthy weight management and stress reduction could guide public health strategies and school-based health education initiatives.

## Conclusions

This study demonstrated a significant negative association between perceived stress and menarcheal age among Al-Madinah adolescent females, with higher stress linked to earlier menarche onset. The findings contribute to regional literature on adolescent reproductive health and reveal complex relationships between stress, obesity, and reproductive development. These results highlight the need for integrated health approaches addressing both physical and psychosocial factors in Saudi Arabia.
